# 

**Published:** 1902-07-15

**Authors:** 


					﻿THE
DENTAL REGISTER
EDITED BY
NELVILLE S. HOFF, D. D.S.,
ANN ARBOR, MICH.
Vol. LVI.	JULY 15, 1902.	No. 7.
PRICE, ONE DOLLAR /M^A^^FTCSVANCE.
PUBLISHED MONTHLY BY
SAMUEL A. CROCKER & CO.,
THE OHIO DENTAL AND SURGICAL DEPOT.
to 39 ’W. “tli J*»t., Ciiiciiiriiiti, O.
Entered at the Postoffice at Cincinnati, 0., as second-class mail matter.
				

